# Integrating health across the Sustainable Development Goals in the Eastern Mediterranean Region: Assessment of Voluntary National Reviews from 18 countries

**DOI:** 10.1371/journal.pgph.0003451

**Published:** 2024-07-11

**Authors:** Ruth M. Mabry, Henry V. Doctor, Mina N. N. Khair, Maha Abdelgalil, Arash Rashidian

**Affiliations:** 1 Global Health Consultant, Muscat, Oman; 2 Department of Science, Information and Dissemination, World Health Organization Regional Office for the Eastern Mediterranean, Cairo, Egypt; 3 WHO Health Emergency Programme, World Health Organization Regional Office for the Eastern Mediterranean, Cairo, Egypt; PLOS: Public Library of Science, UNITED STATES

## Abstract

Voluntary National Reviews are the formal mechanism for countries to report on progress and share lessons learned on SDG implementation. We assessed the Voluntary National Reviews submitted by countries in the WHO Eastern Mediterranean Region to note the reported progress on Sustainable Development Goal (SDG) implementation, the review preparation process and how health is positioned and framed to identify priorities for accelerating progress on the health-related SDGs. We extracted quantitative and qualitative data from 26 Voluntary National Reviews from 18 countries submitted during the period 2016 to January 2022. We focused on three areas: SDG implementation, the review preparation process and the positioning of health in the reviews. Three assessors conducted the data extraction based on the agreed framework according to their language of expertise (Arabic, English and French). One assessor supervised the whole process for consistency. If there was a doubt in interpretation, it was discussed and agreed among the assessors. Countries have established a mechanism for SDG implementation under high-level leadership, engaged in multistakeholder consultations, aligned and mainstreamed SDGs to national plans, and created a monitoring and review mechanism. Countries reported use of national participatory approach for the report preparation. The prioritization of both health and well-being (SDG3) and economic growth (SDG8) in the reports is unique compared to other regional and global assessments. Health was often framed around disease and health care provision without linkages to societal inequities and structural challenges. The reports indicated good progress in SDG implementation. Addressing upstream issues and the determinants of health requires the health sector to take a more strategic approach in advocating for health and well-being. Further research is needed to demonstrate how to address synergies and trade-offs of policy choices and identify the co-benefits of addressing health in the context of fiscal instability and widening inequities in the region.

## Introduction

WHO constitution defines health as “a state of complete physical, mental and social well-being and not merely the absence of disease or infirmity" and recognizes health as a fundamental right and governments have a responsibility for the health of their people [1]. The Alma Ata Declaration reiterates these principles explaining that primary health care involves all related stakeholders “in particular agriculture, animal husbandry, food, industry, education, housing, public works, communications and other sectors” [2]. This comprehensive approach aligns well with the Sustainable Development Goals (SDGs) where SDG 3 focuses on ensuring healthy lives and promote well-being for all at all ages. SDG3 is a key indicator and evidence of success in meeting the 2030 Agenda [3, 4].

COVID-19 pandemic impacted progress on the SDGs. Health was placed high on the political agenda during the COVID-19 pandemic; a whole-of-government approach was used and lives were saved demonstrating the interdependency of health and well-being with other goals [5]. The synergies between goals is reflected in the five dimensions of sustainable development (people, prosperity, planet, partnership and peace) described in Agenda 2030 [4] and three pillars of sustainable development (well-being, infrastructure and natural development) [6]. Like COVID-19, climate change also demonstrates the need for a more integrated whole of society approach to promote health and well-being. With only seven years to 2030, the SDGs is an opportunity to work for more integrated policy-making to promote health and well-being.

Voluntary National Reviews are the formal mechanism for countries to report on progress and share lessons learned on SDG implementation [7]. Although SDG3 on health and well-being is a key indicator of success, a comparative public health assessment of Voluntary National Reviews from European countries reported that health and well-being is not adequately represented in the review process [8, 9]. Their framework of analysis focused on implementing the European SDGs agenda. These reports called for greater advocacy on the integration of health and well-being across the SDGs by demonstrating how investments in health can produce co-benefits to support progress on other SDGs [8, 9]. This is the first assessment of Voluntary National Reviews submitted by countries in the WHO Eastern Mediterranean Region (EMR) to be done from a public health perspective. Our assessment broadens the scope of analysis compared to the European reviews. We assessed progress on SDG implementation, the reviews’ preparation process and how health is positioned and framed in the reviews to identify priorities for accelerating progress on the health-related SDGs.

## Methods

We reviewed the latest Voluntary National Reviews from 18 countries of the EMR submitted from 2016 and January 2022 and available on the UN website (Table 1) (https://hlpf.un.org/countries). Previous reviews of Afghanistan, Egypt, Iraq, Morocco, Qatar, and Tunisia were also assessed, when available. The assessment covered three areas: SDG implementation, the reviews’ preparation processes and the positioning of health and well-being in the reviews. Assessing SDG implementation covered 10 areas proposed by Allen and colleagues: governance and coordination mechanisms including level of leadership, conduction of multistakeholder consultations, SDG mapping and alignment with national strategies and plans, prioritization and adaptation of targets and indicators, mainstreaming the SDG agenda into existing/new strategies, development of an SDG roadmap or action plan, assessment of interlinkages between SDGs, policy evaluation and design, and monitoring and review arrangements [10].

**Table 1:** Status of SDG Voluntary National Reviews in Countries of the Eastern Mediterranean Region *(as of 4 January 2022)*.

Countries	2016	2017	2018	2019	2020	2021	Total
Afghanistan		x				x	2
Bahrain			x				1
Djibouti							0
Egypt	X		x			x	3
Iran							0
Iraq				x		x	2
Jordan		x					1
Kuwait					x		1
Lebanon				x			1
Libya^a^					x		1
Morocco^b^	x				x		2
Occupied Palestinian territories			x				1
Oman				x			1
Pakistan				x			1
Qatar		x	x			x	3
Saudi Arabia			x				1
Somalia							0
Sudan			x				1
Syria^a^					x		1
Tunisia^b^				x		x	2
United Arab Emirates	x						1
Yemen							0
**Total**	**3**	**3**	**6**	**5**	**4**	**5**	**26**

Note: All reports available in English except those marked

^a.^Arabic

^b^French; Djibouti and Somalia submitted their reviews in September 2022; Iran and Yemen has not yet submitted a Voluntary National Review based on the UN website.

We assessed the reviews preparation process to see how well the country reports adhered to the principles in the UN voluntary review guidelines [7] including: country ownership, track progress, report challenges, use a participatory approach, report on gender-and equity-responsiveness, build on existing platforms, use of country-led assessments and evaluations, the review related capacity building and UN support. Tracking progress involved assessing SDGs prioritization and reporting of targets and indicators across income groups using World Bank classifications (low- middle- and high-income countries, respectively; see: https://data.worldbank.org/) (Low income: Afghanistan, Sudan, and Syria, Middle income: Egypt, Iraq, Jordan, Lebanon, Libya, Morocco, occupied Palestinian territory, Pakistan, and Tunisia, High income: Bahrain, Kuwait, Oman, Qatar, Saudi Arabia and United Arab Emirates), five dimension of sustainable development (people, prosperity, planet, partnership and peace) [4] and three pillars of sustainable development (well-being, infrastructure and natural development) [6].

We adapted the framework used in the European region to see how well health was positioned in SDG implementation and framed in the submitted reviews [8]. We assessed the alignment of health and well-being in the national development plan, commitments to four health-related international agreements (Framework Convention of Tobacco Control—FCTC, International Health Regulations—IHR, Sendai and the Paris Agreement, and the prioritization of SDG3.

Spreadsheets were developed based on the three areas to be assessed and used to extract data and facilitate analysis. Three assessors conducted the data extraction based on the agreed framework and reviewed the reports available in Arabic, English and French according to their language of expertise. One assessor supervised the whole process for consistency. If there was a doubt in interpretation, it was discussed and agreed among the assessors. Both qualitative and quantitative analysis were used. Quantitative analysis involved tallying the results using Excel. Qualitative analysis involved thematic analysis of the data extracted to identify common topics and concepts. The Standards for Reporting Qualitative Research, a 21-item checklist for transparent reporting on all aspects of qualitive research, was followed to assist with manuscript preparation [11].

## Results

### SDG implementation

All countries reviewed have established a governance mechanism (Table 2); heads of states oversee its implementation in seven countries (Egypt, Jordan, Lebanon, Morocco, Occupied Palestinian territory (oPt), Pakistan, and Sudan). All countries have held multistakeholder consultations and developed a monitoring and review process; most have conducted an SDG mapping exercise to ensure its alignment with national development strategies and plans. Jordan and Sudan are the only two countries who created an SDG roadmap; the former explicitly aims to build SDG “ownership and achievement.” Few countries have conducted assessments linking SDGs such as using the United Nations Development Programme (UNDP) Rapid Appraisal Instrument [12] (Afghanistan, Iraq, Morocco and Saudi Arabia) or thematic assessments to guide policy action and prioritization of SDG targets. For example, Egypt, with support from UNICEF, examined the determinants of poverty and Sudan conducted a stakeholder assessment of national policies which resulted in developing a roadmap for the implementation of a Health in All Policies approach.

**Table 2:** Progress in SDG implementation, Voluntary National Review preparation process, and health and well-being in the Eastern Mediterranean Region, 2016–2021.

Country, year	Progress on SDG implementation										Voluntary National Review preparation process										Health and well being		
	Governance mechanism	Head of governance mechanism	Multistakeholder consultations	SDG mapping and alignment	Prioritise/adapt targets	Mainstreaming SDGs or creating a separate plan	SDG roadmap	Assess interlinkages	Policy evaluation	Monitoring and review mechanisms	Country ownership	Participatory	Track progress	Challenges	Gender-sensitive	Equity-sensitive	Existing platforms	Assessments	Capacity building	UN Support	Alignment of health in national development plan	International Health Agreements^e^	Prioritizing health and well-being (SDG3)
Afghanistan, 2021		Minister				Existing																P	
Bahrain, 2018		Minister				Existing																SP	
Egypt, 2021		Head of State				Existing																	
Iraq, 2021		Minister				Existing																	
Jordan, 2017		Head of State				Existing																P	
Kuwait, 2019		Minister				Existing																P	
Lebanon, 2018		Head of State				Existing																P	
Libya, 2020		Minister				Existing																	
Morocco, 2020		Head of State				Existing																ISP	
oPt, 2018		Head of State				Existing																P	
Oman, 2019		Minister				Existing																FIP	
Pakistan, 2019		Head of State				New																IP	
Qatar, 2021		Minister				Existing																	
Saudi Arabia, 2018		Minister				Existing																P	
Sudan, 2018		Head of State				Existing																	
Syria, 2020		Minister				Existing																	
Tunisia, 2021		Minister				Existing																ISP	
UAE, 2016		Minister				Existing																SP	

Note: Black shaded cells denote step, approach or guideline applied, ^a^International Agreements include F (Framework Convention on Tobacco Control, I (International Health Regulations, 2005), S (Sendai Framework on Disaster Risk Reduction, 2015–2030) and P (Paris Agreement); oPt: occupied Palestinian territory, UAE: United Arab Emirates

### Voluntary National Review preparation processes

Although only two countries (Afghanistan and Egypt) adhered to all ten principles in the preparation processes, all countries had a national high-level team leading the preparations, used a participatory approach and utilized existing platforms in preparing the report (Table 2). The review preparation processes in most countries engaged at least the line ministries, the private sector and civil society organizations (S1 Table). Eight countries included government institutions beyond line ministries such as financial institutions, academia, and national councils (Afghanistan, Bahrain, Egypt, Morocco, Oman, Qatar, Saudi Arabia and Tunisia); Pakistan was the only country that mentioned engaging sectors at the sub-national level. In addition to civil society organizations, several countries mentioned engaging additional institutions and individuals such as members of parliament, think tanks and representatives from vulnerable groups. United Nations engagement in the review preparation processes varied significantly from no involvement (Bahrain, Pakistan, Qatar, Saudi Arabia, and the United Arab Emirates to 21 agencies in Jordan (S2 Table). The UNDP was the most frequently mentioned UN agency in the reviews. The WHO was only involved in the preparation of four reviews (Egypt, Jordan, Morocco, and Tunisia).

The progress of SDGs was usually presented in numerical order with only 6 countries (Bahrain, Jordan, Lebanon, occupied Palestinian territory (oPt), Sudan and Syria) grouping the goals around the five dimensions of sustainable development (5 Ps: people, prosperity, planet, partnership and peace) [4] when reporting progress on specific goals. Many reports expressed political commitment to gender equality and equity. More than half of the countries (Afghanistan, Egypt, Iraq, Jordan, Kuwait, Lebanon, Libya, oPt, Qatar, Syria and Tunisia) presented sex-disaggregated data beyond SDG5 on gender equality and/or shared examples of efforts on gender-mainstreaming in policy and programs. Similarly, many reported on inequalities such as discussing the Gini-coefficient, presenting disaggregated data (i.e., rural-urban, wealth, education levels, geographical) and/or engaged representatives from vulnerable populations during the review preparation processes. On the other hand, only a few countries reported about policy evaluations. In addition to the policy assessment mentioned earlier, Egypt conducted an impact assessment for a cash transfer program. Both Lebanon and Pakistan conducted strategic assessment of food and nutrition with support from the World Food Programme.

The two most common challenges to SDG implementation were related to economic development (n = 16) and data availability (n = 12). High dependence on oil in high-income countries, high fiscal deficits and debt ratios and limited fiscal space were common concerns in middle-income countries and the high dependence on development partners and heavy indebtedness were the main economic concerns in low-income countries. The economic downturn prior to the pandemic and the economic impact of the pandemic were mentioned. Although limited data availability and statistical capacity were major concerns, only seven countries presented an analysis on data availability. Weak governance for effective coordination and policy coherence, regional/national conflict and instability and environmental challenges including climate change and high dependence on fossil fuels were other challenges commonly mentioned.

**Fig 1:**
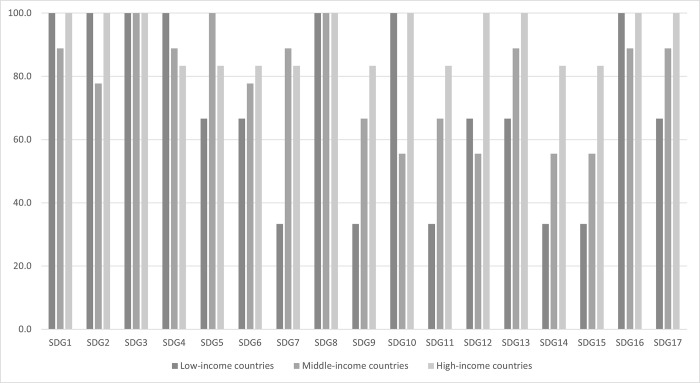
Proportion of countries prioritizing SDGs, by income levels about here. Note for Fig 1. Low-income countries: Afghanistan, Sudan, and Syria, Middle-income countries: Egypt, Iraq, Jordan, Lebanon, Libya, Morocco, occupied Palestinian territory, Pakistan, and Tunisia, High-income countries: Bahrain, Kuwait, Oman, Qatar, Saudi Arabia and United Arab Emirates.

**Fig 2:**
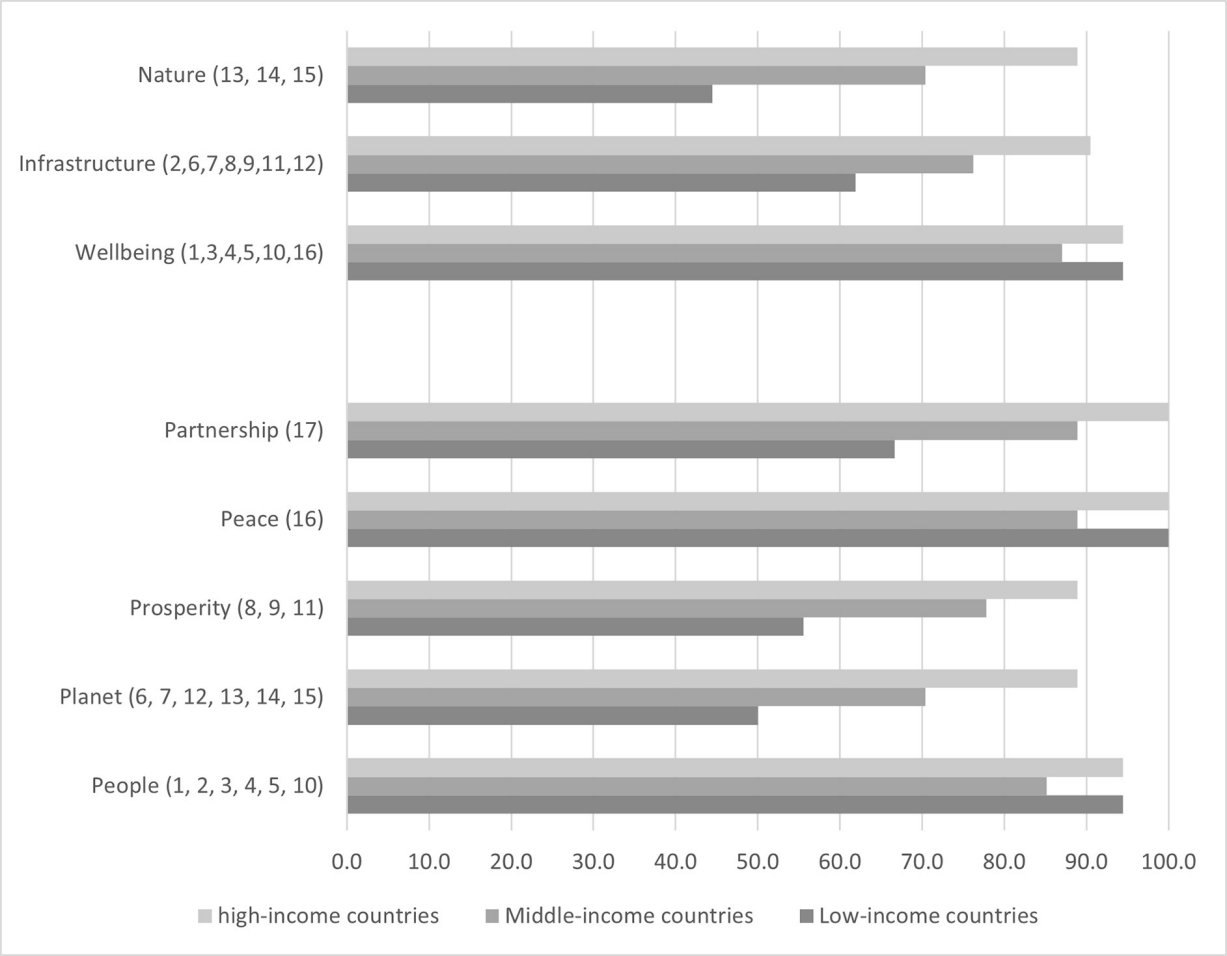
Proportion of reported SDGs in the Eastern Mediterranean Region according to the 5 dimensions of sustainable development and 3 sustainable pillars, by income, 2016–2021 about here. Note for Fig 2: Numbers in parenthesis denote the SDGs; Low-income countries: Afghanistan, Sudan, and Syria, Middle income countries: Egypt, Iraq, Jordan, Lebanon, Libya, Morocco, occupied Palestinian territories, Pakistan, and Tunisia, High income countries: Bahrain, Kuwait, Oman, Qatar, Saudi Arabia and United Arab Emirates].

Reporting on SDG 3 and SDG 8 on economic growth were prioritized in all the reviews of the region. Eleven reviews reported on all 17 SDGs; 17 reported on SDG1 on poverty and SDG 16 on peace. SDG 14 and 15 on life below water and life on land (n = 11) were the least common reported (Fig 1, Table 3). When grouped according to their income levels, high-income countries prioritized a higher proportion of SDGs (92·2%), compared to middle-income (79·1%) and low-income countries (70·6%). All high-income countries and low-income countries reported on SDG1, SDG2, SDG10 and SDG 16, but this varied for middle-income countries (88.9%, 77·8%, 55·6% and 88·9% respectively). The widest divergence in reporting were for SDG9, 11, 14 and 15. When grouping SDGs according to the 5 Ps and three sustainable development pillars [6] People and Peace were the most commonly reported sustainable development dimensions and Wellbeing the most common sustainable development pillar across all income groups. The widest divergence is in Planet and Prosperity dimensions and the Nature pillar (Fig 2).

**Table 3:** SDGs prioritized in the Voluntary National Reviews in the Eastern Mediterranean Region, 2016–2021.

Country (year)	SDG1	SDG2	SDG3	SDG4	SDG5	SDG6	SDG7	SDG8	SDG9	SDG10	SDG11	SDG12	SDG13	SDG14	SDG15	SDG16	SDG17	Total
Afghanistan, 2021	x	x	x	x	x	x		x		x		x	x			x	x	12
Bahrain, 2018	x	x	x	x	x	x	x	x	x	x	x	x	x	x	x	x	x	17
Egypt, 2021	x	x	x	x	x	x	x	x	x	x	x	x	x	x	x	x	x	17
Iraq, 2021	x		x	x	x			x								x	x	7
Jordan, 2017	x	x	x	x	x	x	x	x	x				x			x		11
Kuwait, 2019	x	x	x	x	x	x	x	x	x	x	x	x	x	x	x	x	x	17
Lebanon, 2018	x	x	x	x	x	x	x	x	x	x	x	x	x	x	x	x	x	17
Libya, 2020			x	x	x	x	x	x			x		x			x	x	10
Morocco, 2020	x	x	x	x	x	x	x	x	x	x	x	x	x	x	x	x	x	17
oPt, 2018	x	x	x	x	x	x	x	x	x	x	x	x	x	x	x	x	x	17
Oman, 2019	x	x	x	x	x	x	x	x	x	x	x	x	x	x	x	x	x	17
Pakistan, 2019	x	x	x		x		x	x					x				x	8
Qatar, 2021	x	x	x					x		x		x	x			x	x	9
Saudi Arabia, 2018	x	x	x	x	x	x	x	x	x	x	x	x	x	x	x	x	x	17
Sudan, 2018	x	x	x	x				x		x						x		7
Syria, 2020	x	x	x	x	x	x	x	x	x	x	x	x	x	x	x	x	x	17
Tunisia, 2021	x	x	x	x	x	x	x	x	x	x	x	x	x	x	x	x	x	17
UAE, 2016	x	x	x	x	x	x	x	x	x	x	x	x	x	x	x	x	x	17
Total	15	14	16	14	14	12	12	16	10	12	10	11	14	9	9	15	14	

Note: oPt: occupied Palestinian territories; UAE: United Arab Emirates

### Framing of health in the Voluntary National Reviews

Although 12 reviews explicitly confirmed the alignment of health in the national development plan (Table 2), only two countries described the extent of health sector engagement across development plans. In Jordan, the SDG mapping exercise showed how SDG3 linked to eight of 18 working groups. Pakistan’s Annual Plan, 2019–2020 included 19 strategies with SDG3 mapped to seven of them. As a line ministry, the health sector was engaged in the preparation of all the reviews. The reports from Afghanistan and Bahrain were the only ones that mentioned that the health sector was engaged in reporting beyond SDG3; although this may be the case in other countries, it was not explicitly mentioned.

All reviews reported on SDG3 (Table 2 and Fig 1), however, reporting on the progress on SDG3 was mostly limited to progress on national indicators using a biomedical approach that focuses on diseases, health conditions and service provision. Reports structured around the five sustainable development dimensions or submitted during the COVID-19 pandemic era (Afghanistan, Egypt, Iraq and Qatar) where the societal and health impact and multisectoral government response is described, reporting on progress on SDG3 was similar. In other words, few reports examined structural challenges, health inequities or determinants of health.

For example, maternal mortality ratio (MMR), the only indicator mentioned in all reviews, almost all reports presented national figures. Some reports presented trends and/or disaggregated indicators. Morocco’s report attributed improvements in the MMR to improved quality of care and skilled birth attendance while Pakistan’s described their plan to increase skilled birth attendance. Jordan’s report went further by stating that “further improvement will depend on better targeting of more vulnerable cases and a reduction in disparities in the quality of health care provided to all women of child-bearing age.” Three reports made links to other SDGs. Sudan’s review, the only one framed around 3 themes of the country’s transformation agenda (peace, agriculture transformation and social transformation), mentioned that the implementation of nutrition policies would “improve the health status of infants, children and their mothers.” Oman’s review correlated girl’s education to increased average age at first marriage and decline in adolescent birth rate; however, the text does not link these trends directly with the decline in maternal mortality. Syria’s linked the increase of MMR to the current crisis and absence of skilled birth attendance and explained their focus on addressing determinants like water quality and nutrition.

Although 12 countries mentioned the Paris Agreement (Table 2), few mentioned other health-related international treaties (IHR: Morocco, Oman, Pakistan, Qatar, Tunisia and United Arab Emirates, Sendai Framework: Bahrain, Morocco and Tunisia and FCTC: Oman). The IHR, where the health sector would lead implementation, Tunisia’s review was the only review that not only reported on the indicator but elaborated on actions taken based on the recommendations of the 2016 Joint External Evaluation [13]: the country adopted the food safety law, developed a national biosafety, biosecurity strategy and a prevention, preparedness, response and resilience plan of action towards diseases with epidemic potential.

## Discussion

Our study of Voluntary National Reviews from 18 EMR countries, seven years since the launch of Agenda 2030, found that progress has been made in key areas of SDG implementation in the region. The areas of progress included establishing a mechanism for SDG implementation under national leadership, engaging in multistakeholder consultations, and mainstreaming SDGs to national plans. Countries largely adhered to the UN guiding principles when preparing their reviews, while only two countries adhered to all the principles. All countries reported on SDG3 (health) and SDG8 (economic growth); poverty and peace were other key SDGs across most countries with much greater divergence on prioritization of other SDGs and by income levels. Despite SDG3 prioritized for all countries, it was simplistically framed around disease and health care provision.

We also noted that only eight countries made efforts to map SDG targets with national plans and analyse interlinkages between goals and targets to guide decision-making processes [12]. This was a key consideration and may explain some of the delays in achieving SDGs, for example in health areas, although the factors behind it are more complex [14]. Remarkably only two countries engaged UN country teams in the preparation of the reviews, a key finding that needs to be addressed by better engagement of the UN teams at national level. Expectedly, for the region, we noted a majority of countries gave ample attention to conflicts, emergencies and migration related polices and priorities SDG16 [15, 16]. We noted that most of the 12 EMR countries affected by armed conflicts had submitted at least one review. By contrast, attention to environmental threats to livelihood and development in the countries was not a dominant feature in the reports.

We noted that the attention to equity aspects in the reports was far from ideal. Although the reviews articulated governments’ commitment to the 2030 Agenda and the equity related goals under the SDGs, only half the reports provided some information on inequities, generally focused more on overall goals. Limited availability of data and potential analytical capacity in the countries were potential causes behind this, as also noted elsewhere [17].

Few previous studies have assessed the voluntary national reviews in a systematic way from a health perspective [8, 9, 18], and our study adds to this body of knowledge. The importance of such multi-country studies is in their value in going beyond one country approach and examine the wider UN interaction with the countries and reflect on how countries are addressing their commitments mandated under UN umbrella. Our study is the first such study in the EMR region, and as such adds further value for better governance mechanisms for the monitoring of SDGs implementation and help UN’s regional structures for enhancing collaboration in key areas, including health, economic growth and peace.

The clear prioritization of SDG3 and SDG8 in the reviews stands in sharp contrast to the review of reports from 17 countries in Latin America where most countries reported on poverty eradication (SDG1) and health [19] and the review of 19 countries of varying income levels found that poverty eradication (SDG1) and economic growth were more consistently reported [20]. Health is at the centre of Agenda 2030; success on other goals impacts the health and well-being of a population [21]. Investing in health, particularly in addressing gaps revealed by the pandemic, requires putting health and well-being at the core of government policy making [22]. Despite the priority placed on health in the reviews, in reality, health is not a priority as government spending on health as a share of overall spending is lower (7.6%) than the global average (10%) including among the high-income countries [23]. The health sector could take advantage of the SDG collaborative structure to promote a more holistic approach for optimizing co-benefits for all sectors, ensure policy coherence, and effective use of resources [24–26]. An example of such approach was Sudan’s pre-crisis experience with Health in All Policies in which the contribution all sectors to health and its determinants is better accounted for and owned by the responsible governmental offices [9, 24, 25]. United Arab Emirates’ national program for happiness and positivity is another prominent example of how integrated policy approaches can better address interlinked SDGs goals.

Economic growth as a priority and a concern across most reports points to the on-going challenges of ensuring sustainable economic growth by the countries of the region that undermine fiscal stability and widen inequities [27]. The paradigm shift embraced by the SDG agenda requires a shift from focusing on economic growth and to one that prioritizes health and well-being and promotes intersectoral action and prevention [21, 26, 28–32].

Bringing about a system transformation as envisaged by Agenda 2030 requires strengthening governance mechanisms that support greater policy coherence across SDGs, another weakness commonly mentioned across most the reviews. Promoting health and well-being depends on actions beyond the remit of ministries of health. It requires multisectoral action where health professionals are “champions for health” [8] going beyond their technical expertise to a broader health approach like impact assessments and economic evaluations [9]. It requires strong governance mechanisms and processes that facilitate all stakeholders to discuss interests and responsibilities to reach a common approach [33]. Rasanathan and colleagues propose ten key strategies for multisectoral action including but not limited to understanding key actors and political ecosystems, framing the issue in the most strategic manner, defining clear roles with specific sets of interventions according to sector, and addressing conflicts of interest [34]. Promoting policy coherence for health and well-being for all can also build on existing partnerships and platforms such as the Eastern Mediterranean Regional Healthy Cities Network [8, 35].

Further collaboration between the UN agencies is key under the Global Action Plan for Healthy Lives and Well-being [36, 37]. The Regional Health Alliance, a WHO lead partnership of 17 health and development partners, provides an important platform to enhance coordination and collaboration and leverage regional resources and opportunities for joint initiatives of the Global Action Plan for Healthy Lives and Wellbeing [38]. UN agencies, health and development partners and funding agencies could support addressing gaps identified in this review such as assessing interlinkages across targets, conducting policy evaluations, improving data availability, and building national capacity. However, countries should take the lead in bringing them together to ensure alignment with national priorities [39].

Our study suffers from some limitations. Voluntary National Reviews for the SDGs are highly formalized country reports that attempt to address expectations from all sides and address country response toward the SDGs. Thus, a study based in these reviews may not provide comprehensive information regarding the actions taken for specific SDGs or actions at the sub-national and local levels which may not be included in the national reports. The reviews may also contain a bigger challenge. In many countries, the national data may not be adequately cover the key indicators that demonstrate the national outcomes or the variables that affect equity (e.g. gender, education, socioeconomic status, place of living and ethnicity). Hence the voluntary reviews may not provide adequate information for in-depth understanding of the country needs or priorities [22, 24, 40]. One assessor carried out the data extraction from each review which may have resulted in missing information. Also we did not share the extracted data with countries nor sought feedback from them which limits the accuracy. The aim was to keep this content analysis focused on the content presented in the lengthy Voluntary National Reviews as key policy documents. When in doubt in the comprehensiveness of a country report, we adopted a more positive interpretation so any bias would be on the optimistic side.

The findings from this review point to the need to use the observed progresses or challenges toward SDGs and policy and economic evaluations to guide policy action and promote policy coherence for health and well-being [10]. Interlinkages between goals including efforts addressing determinants of health, health inequities and upstream factors affecting health are missing across many Voluntary National Reviews. Building the evidence to fill these gaps to address synergies and trade-offs of policy choices, especially showcasing the co-benefits of addressing health to achieving the 2030 Agenda, is key to shaping interventions to address national priorities [41–43]. It would also provide the basis for the transformation called for by Agenda 2030 and the paradigm shift towards a sustainable economy that prioritizes health and well-being [30].

## Conclusion

The Voluntary National Reviews reported good progress made in SDG implementation in countries of the region. The prioritization of both health and well-being (SDG3) and economic growth (SDG8) is unique in comparison to other regional and global reviews. Addressing upstream issues and the determinants of health, such as using the health in all policy framework, requires the health sector to take a more strategic approach in advocating for health and well-being. Further research is needed to demonstrate how best to address synergies and trade-offs of policy choices like national assessments of interlinkages across SDGs and policy evaluations and to identify the co-benefits of addressing health, particularly in the context fiscal instability and widening inequities in the region.

## Supporting information

S1 Table Participating entities in the VNR preparation process in the Eastern Mediterranean Region, 2016–2021. (DOCX)

S2 Table UN Agencies engaged in the VNR preparation process in the Eastern Mediterranean Region, 2016–2021. (DOCX)
