# Correction: Integrating health across the Sustainable Development Goals in the Eastern Mediterranean Region: Assessment of Voluntary National Reviews from 18 countries

**DOI:** 10.1371/journal.pgph.0004832

**Published:** 2025-06-18

**Authors:** Ruth M. Mabry, Henry V. Doctor, Mina N. Khair, Maha Abdelgalil, Arash Rashidian

The third author’s name is spelled incorrectly. The correct name is: Mina N Khair. The correct citation is: Mabry RM, Doctor HV, Khair MN, Abdelgalil M, Rashidian A (2024) Integrating health across the Sustainable Development Goals in the Eastern Mediterranean Region: Assessment of Voluntary National Reviews from 18 countries. PLOS Glob Public Health 4(7): e0003451. https://doi.org/10.1371/journal.pgph.0003451.
