# Supplementary material for: Integrating health across the Sustainable Development Goals in the Eastern Mediterranean Region: Assessment of Voluntary National Reviews from 18 countries
Source: PLOS Glob Public Health. 2024 Jul 11;4(7):e0003451. doi: 10.1371/journal.pgph.0003451 (PMC11239013; doi:10.1371/journal.pgph.0003451)
Supplement: S1 Table — (DOCX) [file pgph.0003451.s001.docx]

**S1 Table. Participating Entities in the VNR preparation process**

| **Country (year)** | **Public Sector** | **Others** |
| --- | --- | --- |
| **Afghanistan, 2021** | 42 government entities including ministries, financial institutions, and academia | Private sector, CSOs UN Agencies, Asian Development Bank |
| **Bahrain, 2018** | 21 government entities | Private sector, CSOs, academia |
| **Egypt, 2021** | 21 government entities including ministries, financial institutions, councils for special groups (children, women and persons with disabilities) | 46 private companies, 54 NGOs,  international development partners,  members of Parliament, and  academia. |
| **Iraq, 2021** | Not defined | Private sector, CSOs and academia |
| **Jordan, 2017** | Line ministries | Private sector, CSOs, parliamentarians, representatives from marginalized groups (i.e., refugees) and UN agencies |
| **Kuwait, 2019** | Line ministries | Chamber of Commerce, CSOs,  UN Agencies |
| **Lebanon, 2018** | Line ministries | 20 business representatives and 50 CSOs |
| **Libya, 2020** | Line ministries | UN agencies and international development partners |
| **Morocco, 2020** | Line ministries and other government bodies including financial institutions and academia | CSOs, National Human Rights Council, members of parliament, UN agencies, |
| **Occupied Palestinian territories, 2018** | Line ministries | Private sector, national and international NGOs, academia and UN Agencies |
| **Oman, 2019** | Line ministries and other government bodies | Private sector, CSOs. |
| **Pakistan, 2019** | Line ministries at national and provincial levels | Parliamentarians, private sector civil society, think tanks and academia |
|  |  |  |
| **Qatar, 2021** | Line ministries, other government bodies including financial institutions and academia | No information |
| **Saudi Arabia, 2018** | Line ministries, other government bodies including financial institutions and academia | Private sector, NGOs |
| **Sudan, 2018** | Not clear | Private sector, community-based organizations, parliamentarians, academia and UN agencies |
| **Syria, 2020** | All government institutions | Private sector, CSOs, academia, UN Agencies |
| **Tunisia, 2021** | Line ministries, other government bodies including financial institutions and academia | CSOs, national organizations, and UN agencies, |
| **UAE, 2016** | No information | No information |

Note: CSOs: Civil society organizations, NGOs: Non-governmental organizations
