# Supplementary material for: Integrating health across the Sustainable Development Goals in the Eastern Mediterranean Region: Assessment of Voluntary National Reviews from 18 countries
Source: PLOS Glob Public Health. 2024 Jul 11;4(7):e0003451. doi: 10.1371/journal.pgph.0003451 (PMC11239013; doi:10.1371/journal.pgph.0003451)
Supplement: S2 Table — (DOCX) [file pgph.0003451.s002.docx]

**S2 Table. UN Agencies engaged in the VNR preparation process in the Eastern Mediterranean Region, 2016-2021**

| **Country, year** | **Regional Health Alliance Members** | | | | | | | | | | | | | | | **Additional UN Agencies** | | | | | | | | | | | | | **Total** |
| --- | --- | --- | --- | --- | --- | --- | --- | --- | --- | --- | --- | --- | --- | --- | --- | --- | --- | --- | --- | --- | --- | --- | --- | --- | --- | --- | --- | --- | --- |
|  | **IOM** | **ITU** | **UNAIDS** | **UNDP** | **UNESCO** | **UNFPA** | **UN Habitat** | **UNHCR** | **UNICEF** | **UNIDO** | **UNOCHA** | **UN Women** | **World Bank** | **WHO** | **WFP** | FAO | ILO | IMF | UN SRSG^a^ on violence against children | UNDESA | UNEP | UNESCWA | UNIC | UNODC | UNOPS | UNRC | UNRWA | UNV |  |
| Afghanistan, 2021 |  |  |  | X |  |  |  |  |  |  |  |  |  |  |  |  |  |  |  |  |  |  |  |  |  |  |  |  | 1 |
| Bahrain, 2018 |  |  |  |  |  |  |  |  |  |  |  |  |  |  |  |  |  |  |  |  |  |  |  |  |  |  |  |  | 0 |
| Egypt, 2021 | X |  | X | X | X | X |  | X | X | X |  | X | X | X |  |  | X |  | X | X |  | X |  | X |  | X |  | X | 18 |
| Iraq, 2021 |  |  |  | X |  |  |  |  |  |  |  |  |  |  |  |  |  |  |  |  |  | X |  |  |  |  |  |  | 2 |
| Jordan, 2017 | X |  |  | X | X | X | X | X |  | X | X |  | X | X | X | X | X | X |  |  | X |  | X | X | X | X | X | X | 21^a^ |
| Kuwait, 2019 |  |  |  | X |  |  |  |  |  |  |  |  |  |  |  |  |  |  |  |  |  |  |  |  |  |  |  |  | 1 |
| Lebanon, 2018 |  |  |  | X |  | X |  |  |  |  |  |  |  |  |  |  |  |  |  |  |  |  |  |  |  |  |  |  | 2 |
| Libya, 2020 | X |  |  | X |  | X |  |  | X |  |  |  |  |  | X |  |  |  |  |  |  |  |  |  |  |  |  |  | 5 |
| Morocco, 2020 |  |  |  | X |  | X |  |  |  | X |  | X | X | X |  | X |  | X |  |  | X |  |  |  |  |  |  |  | 9 |
| oPt, 2018 |  |  |  |  |  |  |  |  |  |  |  |  |  |  |  |  |  |  |  |  |  |  |  |  |  |  |  |  | b |
| Oman, 2019 |  |  |  | X |  |  |  |  | X |  |  |  |  |  |  |  |  |  |  |  |  | X |  |  |  |  |  |  | 3 |
| Pakistan, 2019 |  |  |  |  |  |  |  |  |  |  |  |  |  |  |  |  |  |  |  |  |  |  |  |  |  |  |  |  | 0 |
| Qatar, 2021 |  |  |  |  |  |  |  |  |  |  |  |  |  |  |  |  |  |  |  |  |  |  |  |  |  |  |  |  | 0 |
| Saudi Arabia, 2018 |  |  |  |  |  |  |  |  |  |  |  |  |  |  |  |  |  |  |  |  |  |  |  |  |  |  |  |  | 0 |
| Sudan, 2018 |  |  |  | X |  | X |  |  | X |  |  |  |  |  |  | X | X |  |  | X |  |  |  |  |  |  |  |  | 6 |
| Syria, 2020 |  |  |  | X |  | X |  | X | X |  |  |  |  |  |  | X | X |  |  |  |  |  |  |  |  |  |  |  | 6 |
| Tunisia, 2021 |  |  |  | X | X | X |  |  | X | X |  | X | X | X |  | X | X | X |  |  | X |  |  | X |  |  |  |  | 13 |
| UAE, 2016 |  |  |  |  |  |  |  |  |  |  |  |  |  |  |  |  |  |  |  |  |  |  |  |  |  |  |  |  | 0 |
| Total | 3 | 0 | 1 | 12 | 3 | 8 | 1 | 3 | 6 | 4 | 1 | 3 | 4 | 4 | 3 | 5 | 4 | 3 | 1 | 2 | 3 | 3 | 1 | 3 | 1 | 2 | 1 | 2 |  |

Note: oPt: occupied Palestinian territories; ^a^The report mentioned engagement of the UN Country Team (UNCT); this list based on UNCT for 2022 (<https://jordan.un.org/en/about/our-team>); ^b^The report mentioned engagement with UN Agencies but does not provide a list of agencies; the UNCT for 2022 in Palestine includes 22 agencies. <https://unsco.unmissions.org/un-country-team-0#:~:text=The%20United%20Nations%20Country%20Team%20%28UNCT%29%20in%20Palestine%2C,agencies%20operating%20in%20the%20occupied%20Palestinian%20territory%20%28oPt%29>.

FAO: Food and Agriculture Organization, ILO: International Labour Organization, IMF: International Monetary Fund, IOM: International Organization on Migration, ITU: International Telecommunications Union, UN Habitat : United Nations Human Settlements Programme, UN Women: United Nations Entity for Gender Equality and the Empowerment of Women, UNAIDS: Joint United Nations Programme on HIV/AIDS, UNDESA: United Nations Department of Economic and Social Affairs, UNDP: United Nations Development Programme, UNEP: United Nations Environment Programme, UNESCO: United Nations Educational, Scientific and Cultural Organization, UNESCWA: United Nations Economic Commission for Western Asia, UNFPA: United Nations Population Fund, UNHCR : United Nations High Commissioner for Refugees, UNIC: United Nations Information Centre, UNICEF: United Nations Children Fund, UNIDO: United Nations Industrial Development Organization, UNOCHA: United Nations Office for the Coordination of Humanitarian Affairs, UNODC: United Nations Office on Drugs and Crime, UNOPS: United Nations Office for Project Services, UNRC: UN Resident Coordinator, UNRWA: United Nations Relief and Works Agency for Palestinian Refugees in the Near East, UNSRSG: United Nations Special Representative to the Secretary General, UNV: UN Volunteers, WFP: World Food Programme, WHO: World Health Organization
